# On Small-Pox Inoculation

**Published:** 1807-06

**Authors:** 


### *To the Editors of the Medical and Physical Journal.*

GENTLEMEN,

ALTHOUGH an opinion was expressed in your last Journal, that communications on Cow pock were not so desirable as formerly, yet I am induced to address you on a subject very intimately connected with it, in consequence of several instances of the fatality of Small-pox inoculation (no less than five) having been related to me by my acquaintance within these few weeks, all from their own knowledge.

That perfect vaccination is a safe and secure mode of preventing small-pox, is now as clearly proved as any medical fact on record, or as any other fact within the reach of human intellect, which does not admit of mathematical demonstration. Numerous, and I believe I may truly say ALL the medical persons of high respectability in the kingdom, have publicly declared their belief in and approbation of it. The legislature has admitted the fact, by decreeing a reward to that distinguished man who first made us acquainted with its benefits; yet there are men in this town, who with impunity destroy their fellow beings by small-pox inoculation. Now, if five instances of this sort have come to my knowledge within as many weeks, without any inquiry for them, how great must the whole number be? And if five direct victims have so perished, how many more must have suffered from the infection generated by these? Surely, the boldest would be deterred if he could but trace the consequences. Doctor Willan, more than ten years ago, without any view to the late blessed discovery, traced *seventeen cases and eight deaths from* ONE *inoculation*; and if we consider that these these cases must increase in a compound ratio, what a frightful picture is presented to the mind.

That some persons, from a sentiment of piety, should reject every species of inoculation, does not much surprise me; though I would fain tell them, that there are numerous lesser ills for which none scruple to avail themselves of the remedies and preventives which Providence has permitted the intellect of man to develope; and why they should refuse to avail themselves of so great a discovery as this, I cannot perceive. — But with our present knowledge of cow-pox, that any should choose to resort to artificial means for security, and of two modes select the one dangerous to the object on whom it is employed, and highly dangerous to their neighbours, in preference to the one which is perfectly safe to both, is to me quite incomprehensible.

I prize regulated freedom highly indeed; nay, by some, am thought an enthusiast for liberty: but I certainly am no admirer of the liberty of savages. Have we quarantine laws? Have we laws for the punishment of arson? Which in the first case, restrain persons, at however much personal inconvenience or loss of property, from subjecting their fellow-men to the most remote risque of pestilential fever; and in the other from firing a house, though every stick of it, and in it, is the property of the firer: And shall the objection, on the score of liberty, (which I have heard strongly urged) prevail, and prevent a law, restraining people from inflicting and propagating small-pox, the most fatal and destructive of all fevers?

Why should not Coroners make inquiry into the causes and motives of these deaths, so clearly produced by artificial and not by natural means? Whatever excuse there may have been for small-pox inoculation in large towns, on the score of individual security, that time is now certainly past; the same Omniscient Spirit that at one period saw it good to visit mankind with that dreadful plague, small-pox, has now vouchsafed to afford us the means of extirpating it; and I wonder that any should be presumptuous enough to endeavour, by artificial means, to perpetuate the evil.

Why should not Searchers be directed, in their reports for the Bills of Mortality, to distinguish those who die by inoculated small-pox from those who die by the natural disease? Although this would not show the full extent of the evil of small-pox inoculation, yet I am mistaken if it would not show so much, that the public would not long endure endure this baneful practice. It would well become the honourable and benevolent member for Yorkshire, and that same Legislative body, who have acquired immortal honour, by giving the final blow to the abominable African traffic in flesh and blood, to turn their attention to this destructive, fatal, and unnecessary waste of life at home. That too was liberty abused, the liberty of Englishmen doing as they pleased with those who were not Englishmen; the power that abolished the one, will, I trust, ere long, perceive it to be a duty to restrain the other. I am, &c.

